# Comparative Analysis of Gene Regulation by the Transcription Factor PPARα between Mouse and Human

**DOI:** 10.1371/journal.pone.0006796

**Published:** 2009-08-27

**Authors:** Maryam Rakhshandehroo, Guido Hooiveld, Michael Müller, Sander Kersten

**Affiliations:** 1 Nutrigenomics Consortium, Top Institute (TI) Food and Nutrition, Wageningen, the Netherlands; 2 Nutrition, Metabolism and Genomics group, Division of Human Nutrition, Wageningen University, Wageningen, the Netherlands; Ecole Normale Supérieure de Lyon, France

## Abstract

**Background:**

Studies in mice have shown that PPARα is an important regulator of hepatic lipid metabolism and the acute phase response. However, little information is available on the role of PPARα in human liver. Here we set out to compare the function of PPARα in mouse and human hepatocytes via analysis of target gene regulation.

**Methodology/Principal Findings:**

Primary hepatocytes from 6 human and 6 mouse donors were treated with PPARα agonist Wy14643 and gene expression profiling was performed using Affymetrix GeneChips followed by a systems biology analysis. Baseline PPARα expression was similar in human and mouse hepatocytes. Depending on species and time of exposure, Wy14643 significantly induced the expression of 362–672 genes. Surprisingly minor overlap was observed between the Wy14643-regulated genes from mouse and human, although more substantial overlap was observed at the pathway level. Xenobiotics metabolism and apolipoprotein synthesis were specifically regulated by PPARα in human hepatocytes, whereas glycolysis-gluconeogenesis was regulated specifically in mouse hepatocytes. Most of the genes commonly regulated in mouse and human were involved in lipid metabolism and many represented known PPARα targets, including CPT1A, HMGCS2, FABP1, ACSL1, and ADFP. Several genes were identified that were specifically induced by PPARα in human (MBL2, ALAS1, CYP1A1, TSKU) or mouse (Fbp2, lgals4, Cd36, Ucp2, Pxmp4). Furthermore, several putative novel PPARα targets were identified that were commonly regulated in both species, including CREB3L3, KLF10, KLF11 and MAP3K8.

**Conclusions/Significance:**

Our results suggest that PPARα activation has a major impact on gene regulation in human hepatocytes. Importantly, the role of PPARα as master regulator of hepatic lipid metabolism is generally well-conserved between mouse and human. Overall, however, PPARα regulates a mostly divergent set of genes in mouse and human hepatocytes.

## Introduction

The liver plays a major role in the coordination of lipid metabolism. It actively metabolizes fatty acids as fuel and is responsible for triglyceride export via synthesis of very low density lipoproteins. An imbalance between these pathways may lead to triglyceride accumulation and thus hepatic steatosis. Studies in mice have indicated that many aspects of hepatic lipid metabolism are under transcriptional control of the Peroxisome Proliferator Activated Receptor α (PPARα), a transcription factor belonging to the nuclear receptor superfamily. It is well established that impaired PPARα function is associated with hepatic lipid accumulation [1]–[3]. Consequently, synthetic agonists for PPARα are explored for the treatment of non-alcoholic fatty liver disease [4].

Besides PPARα, two other PPARs isotypes are known to exist: PPARβ/δ and PPARγ. The PPARs share a common mode of action that involves heterodimerization with the nuclear receptor RXR, followed by binding to PPAR response elements (PPREs) in target genes [5]. Activation of transcription is induced by binding of ligand, leading to recruitment of specific coactivator proteins and dissociation of corepressors. Expression of PPARα and PPARβ/δ is relatively ubiquitous, whereas PPARγ is mainly expressed in adipose tissue, macrophages and colon [6], [7].

PPARα can be ligand-activated by endogenous agonists, which include fatty acids and fatty acid derivatives such as eicosanoids and oxidized fatty acids, as well as by various synthetic compounds [5], [8], [9]. The latter group induces proliferation of peroxisomes in rodents and are thus referred to as peroxisome proliferators. Peroxisome proliferators encompass a diverse group of chemicals ranging from herbicides and insecticides to industrial plasticisers, halogenated hydrocarbons, and fibrate drugs [10], [11].

Most of the research concerning PPARα has focused on its role in the liver. A wealth of studies performed almost exclusively in mice has revealed that PPARα serves as a key regulator of hepatic fatty acid catabolism (reviewed in [12]). Using PPARα null mice, it has been shown that PPARα is especially important for the adaptive response to fasting by stimulating hepatic fatty acid oxidation and ketogenesis [2], [13], [14]. In addition, PPARα has been shown to govern liver inflammation, lipoprotein metabolism, glucose metabolism, and hepatocyte proliferation [12], [15], [16]. The latter response is known to be specific for rodents [17]. The species-specific effects of PPARα agonists on hepatocyte proliferation and associated hepatocarcinogenesis were ascribed to a number of factors including properties intrinsic to the PPARα protein, conservation and functionality of PPREs in the promoter of target genes, and presence or absence of co-regulators depending on the cellular environment [18].

However, apart from the differential effect on hepatocyte and peroxisome proliferation, it is not very clear whether PPARα has a similar role in mice and humans and to what extent target genes are shared between the two species. Based on the lower expression level of PPARα in human liver compared to mouse liver [19], the functionality of PPARα in human liver has been questioned [20]. This notion has been further reinforced by the limited impact of PPARα agonists on lipid metabolism genes in HepG2 cells [21], which represent the most widely used liver cell culture model.

However, a careful and comprehensive comparative analysis of gene regulation by PPARα between mouse and human hepatocytes has yet to be performed. To fill this gap we systematically compared the effect of activation of the transcription factor PPARα in primary mouse and human hepatocytes using a whole genome transcriptomics approach.

## Results

### PPARa plays an important role in primary human hepatocytes

**Figure 1:**
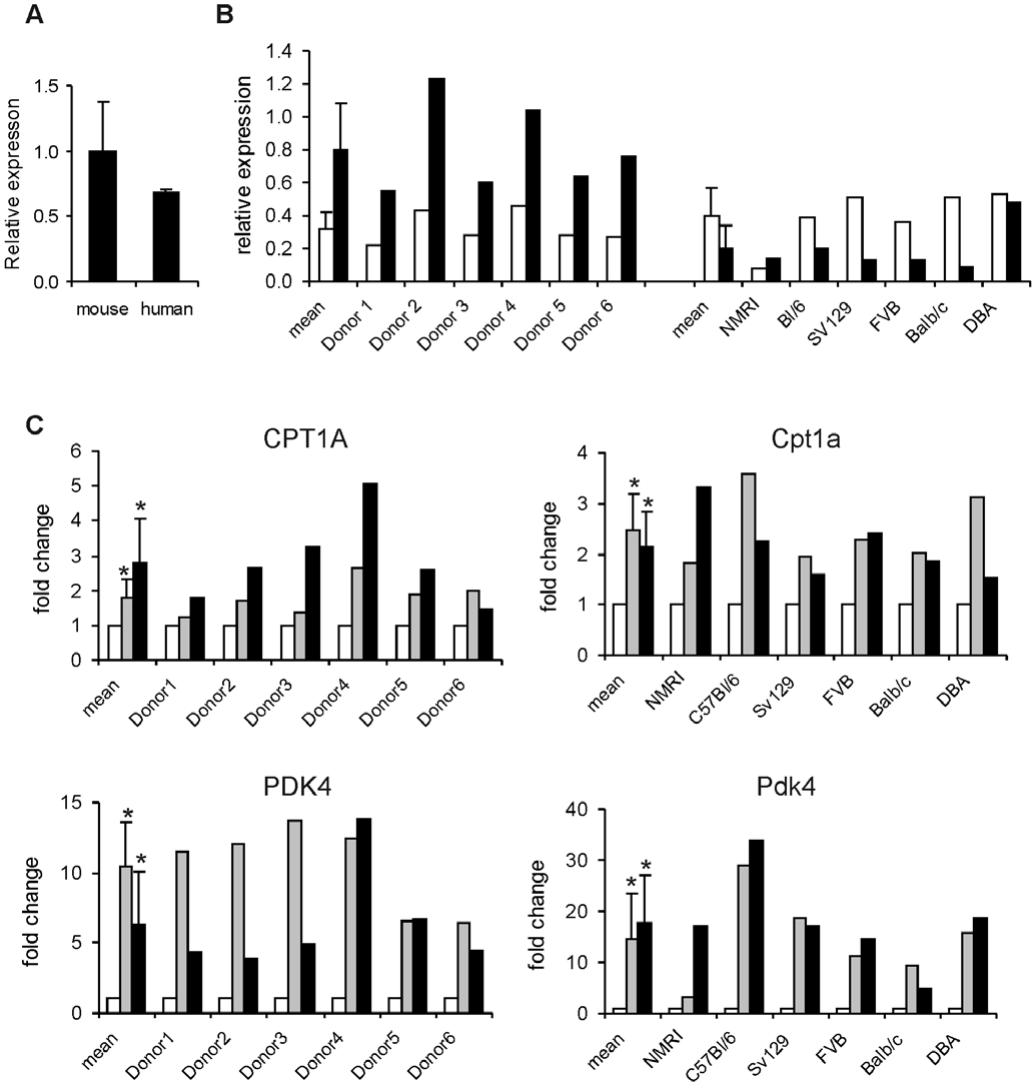
Activation of PPARα in mouse and human hepatocytes. (A) PPARα mRNA expression levels in human versus mouse liver as expressed relative to universal 18S. (B) PPARα mRNA expression levels in human versus mouse primary hepatocytes as expressed relative to universal 18S. Expression was determined at 6 h (open bars) and 24 h (black bars) in control-treated cells (DMSO). (C) Relative induction of expression of carnitine palmitoyltransferase 1A (Cpt1a) and pyruvate dehydrogenase kinase 4 (Pdk4) was determined in human and mouse primary hepatocytes treated with Wy14643 for 6 h (gray bars) and 24 h (black bars). Expression of cells treated with DMSO was set at 1 (white bars). Error bars represent SD. *P<0.05 according to Student's T-test.

We first determined PPARα expression in mouse and human liver by quantitative real-time PCR. PPARα mRNA was only slightly lower in human liver compared to mouse liver (Figure 1A). In order to study the effect of PPARα activation on gene expression in human and mouse liver, primary human and mouse hepatocytes were incubated with the PPARα agonist Wy14643 for 6 or 24 h. To minimize potential statistical bias, the diversity of the six human donors was mimicked by performing the equivalent mouse experiment in primary hepatocytes from six different mice varying in age, sex and genetic background. The choice of using Wy14643 as PPARα agonist was based on a pilot experiment in which primary human hepatocytes were treated with equal concentrations of either Wy14643 or fenofibrate (50 µM). In general, we found that established PPARα target genes were more strongly induced by Wy14643 compared to fenofibrate (data not shown).

The expression of PPARα itself was similar between mouse and human hepatocytes (Figure 1B). While in mouse hepatocytes PPARα mRNA decreased during the course of the incubation, the opposite was the case in human hepatocytes. Treatment with Wy14643 consistently increased the expression of the established PPARα targets Cpt1a and Pdk4 in mouse and human hepatocytes, indicating activation of PPARα (Figure 1C).

To study the effect of PPARα activation on global gene expression, microarray analysis was performed using Affymetrix GeneChips. We first performed principal component analysis (PCA) to sort out the major sources of variation in our microarray data. The PCA plot for 6 h Wy14643 treatment clearly shows that the principal source of variation is between the species (Figure S1). Additionally, the results indicate that: 1) there is large variation between the various mice at basal level (untreated cells), whereas the variation between the human donors is small; 2) the effect of PPARα activation is more pronounced in mice than in humans; 3) the effect of PPARα activation is consistent between the various mice.

**Figure 2:**
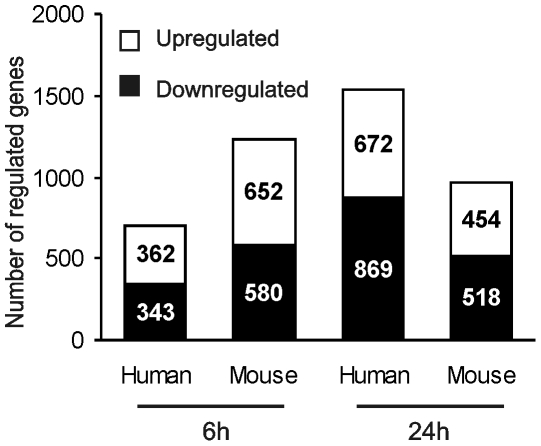
Wy14643 treatment causes major changes in gene expression in human and mouse hepatocytes. Bars show number of up- and down-regulated genes in primary human and mouse hepatocytes treated with Wy14643 for 6 h or 24 h. Genes were considered significantly regulated if mean fold change (MFC)>1.1 and P<0.05.

We found that in human hepatocytes Wy14643 treatment for 6 h significantly altered the expression of 705 genes. A considerably larger number of genes were regulated by Wy14643 in mouse hepatocytes (Figure 2). More stringent selection dramatically reduced the number of significantly regulated genes in human hepatocytes, while it had much less of an effect in mouse hepatocytes (data not shown). Surprisingly, more prolonged Wy14643 treatment augmented the number of significantly regulated genes in human hepatocytes, but not in mouse hepatocytes. The latter result may be related to the lower expression of PPARα in mouse hepatocytes after prolonged incubation (Figure 1B). Overall, these data demonstrate a major impact of PPARα activation in human hepatocytes. All microarray results have been deposited in to the Gene Expression Omnibus (http://www.ncbi.nlm.nih.gov/projects/geo/) and can be accessed online under series number GSE17254.

### PPARa regulates a mostly divergent set of genes in mouse and human primary hepatocytes

**Figure 3:**
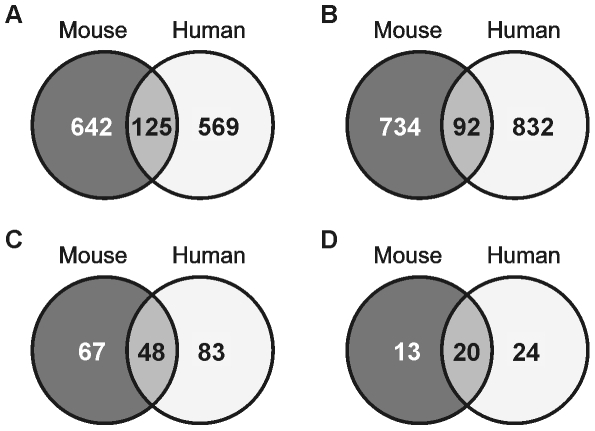
Limited overlap at individual gene level but major overlap at pathway level. Venn diagrams showing overlap in significantly upregulated (A) and (B) downregulated genes after treatment with Wy14643 in mouse versus human hepatocytes. Genes were included if they were significantly regulated by Wy14643 at 6 h and/or 24 h. Criteria for significance was mean fold-change (MFC)>1.1 and P<0.05. Genes without orthologs in the other species and/or not present on the array of the other species were excluded. (C) Venn diagram showing overlap in overrepresented Gene Ontology classes upon Wy14643 treatment in mouse and human hepatocytes based on a functional class score method. Data from 6 h and 24 h Wy14643 treatment were combined in a single analysis. Only GO classes containing 8 to 125 genes and FDR<0.0001 were included in the Venn diagram. (D) Venn diagram showing overlap in upregulated processes analyzed by GSEA. Only gene sets having a size between 15 and 250 genes were included in the analysis. To account for multiple hypothesis tasting, gene sets having a FDR<0.25 were selected. Sources of the gene sets: BIOCARTA, GENMAPP, KEGG, SIGNALING ALLIANCE, SIGNALING TRANSDUCTION, GEARRAY and SK manual.

We next determined the overlap in genes regulated by Wy14643 in mouse and human hepatocytes. Data from 6 h and 24 h Wy14643 treatment were combined to prevent creating a bias from possible differences in kinetics of gene regulation between mouse and human and separate analyses were carried out for up- and down-regulated genes. A total of 125 genes were found to be induced by Wy14643 in both species, many of which were involved in various aspects of lipid metabolism (Figure 3A). A smaller number of genes was found to be downregulated by Wy14643 in both species (Figure 3B). However, the far majority of genes were regulated specifically in one of the species, which would suggest that in general PPARα -dependent gene regulation is poorly conserved between mouse and human. A complete list of regulated genes in the various categories is available in Table S1.

To explore the possible functional impact of PPARα activation in mouse and human hepatocytes, we analyzed for overrepresented Gene Ontology classes in response to Wy14643 treatment using ErmineJ. Again, data from 6 h and 24 h Wy14643 treatment were combined. Out of 115 GO classes overrepresented after Wy14643-treatment of mouse hepatocytes, 48 showed overlap with human hepatocytes (Figure 3C). The overlapping GO classes generally represented various aspects of hepatic fatty acid metabolism including peroxisomal metabolism (Table S2). The GO classes specific for the mouse hepatocytes also mostly corresponded to lipid metabolic pathways, suggesting that regulation of lipid metabolism is the dominant function of PPARα in mouse hepatocytes. In contrast, the GO classes specific for human hepatocytes included alternative metabolic processes including bile acid metabolic process, and various aspects of amino acid metabolism.

**Figure 4:**
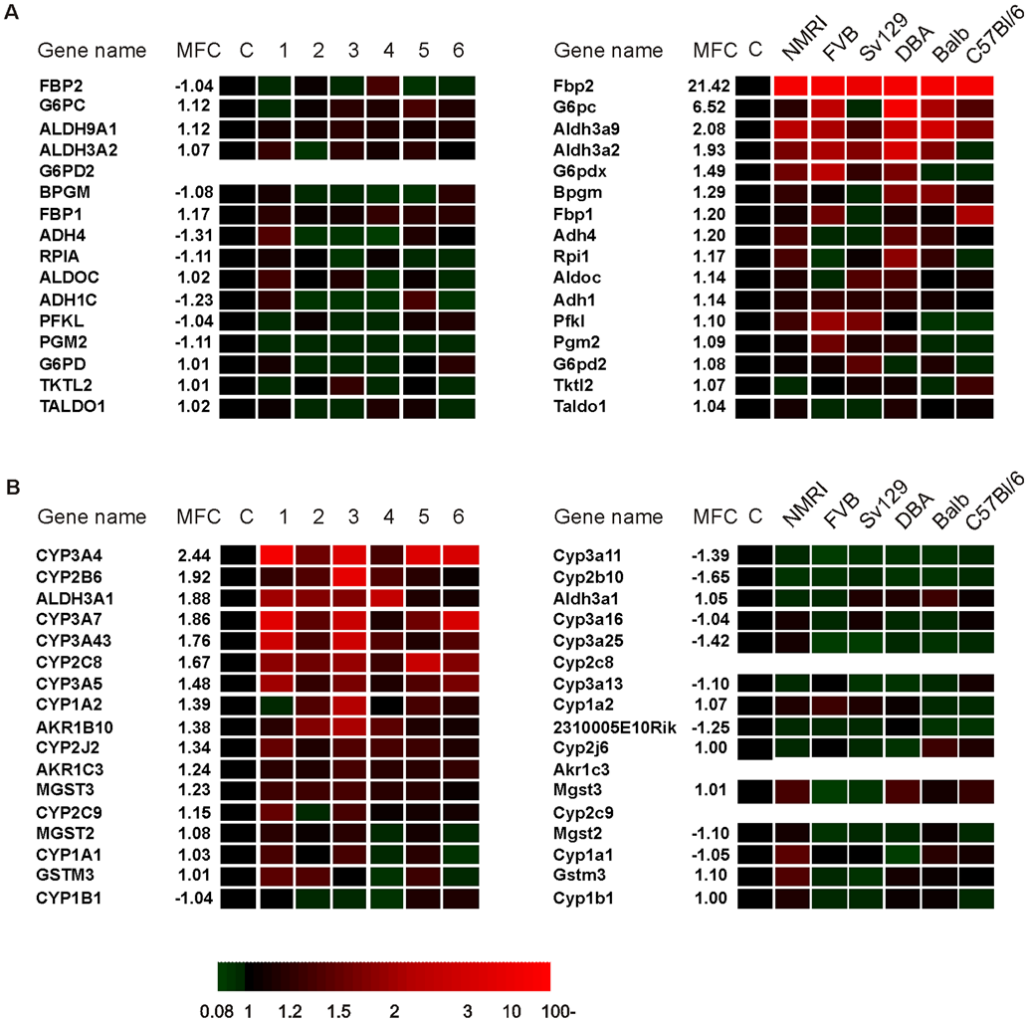
Heat map illustrating the species-specific regulation of two gene sets originating from Gene set enrichment analysis (GSEA). (A) Glycolysis-gluconeogenesis as a mouse-specific upregulated gene set. (B) Xenobiotic metabolism as a human-specific upregulated gene set. Genes are ranked based on the mean fold change (MFC). Expression levels in the DMSO-treated cells were set at 1.

A similar type of analysis focusing on upregulated genes was carried out using gene set enrichment analysis (GSEA). Results from Ingenuity were generally concordant with GSEA and will not further be elaborated on here. Out of 33 pathways induced by PPARα activation in mouse hepatocytes, 20 were also induced in human hepatocytes (Figure 3D), Similar to GO analysis, pathways commonly regulated in mouse and human were mostly related to lipid metabolism (Table S3). Interestingly, the glycolysis-gluconeogenesis pathway was specifically upregulated by Wy14643 in mouse (Figure 4A), while xenobiotic metabolism was specifically upregulated in human (Figure 4B). Overall, these data show that PPARα governs a mostly divergent set of genes in mouse and human hepatocytes, although more significant overlap was observed at the pathway level.

### Identification of human and mouse-specific novel putative PPARα target genes

**Figure 5:**
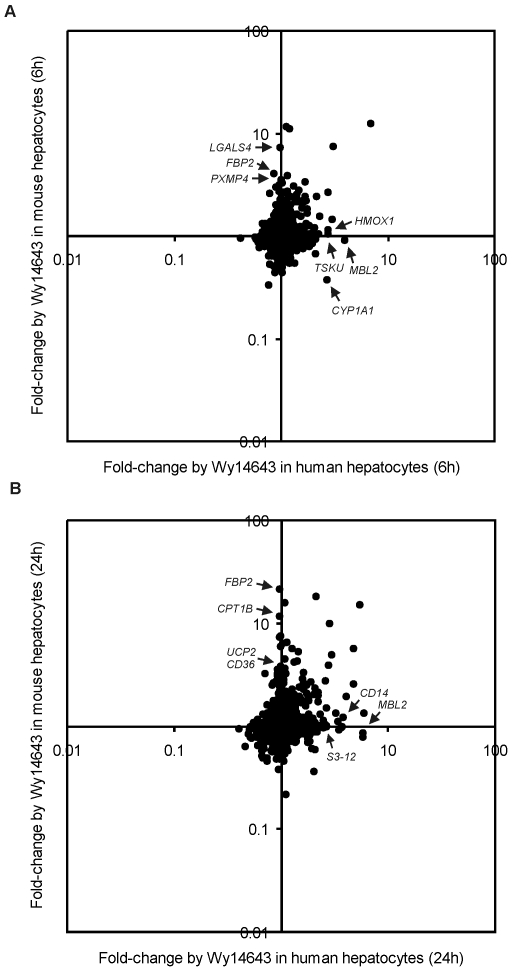
Limited similarity in Wy14643-induced gene regulation between mouse and human hepatocytes. Scatter plots demonstrating similarities and differences in gene regulation by 6 h (A) and 24 h (B) PPARα activation between human and mouse hepatocytes. Graphs show fold-changes in gene expression after treatment with Wy4643 in human hepatocytes (x-axis) and mouse hepatocytes (y-axis). Selected genes that are upregulated specifically by Wy4643 in human or mouse are indicated.

**Figure 6:**
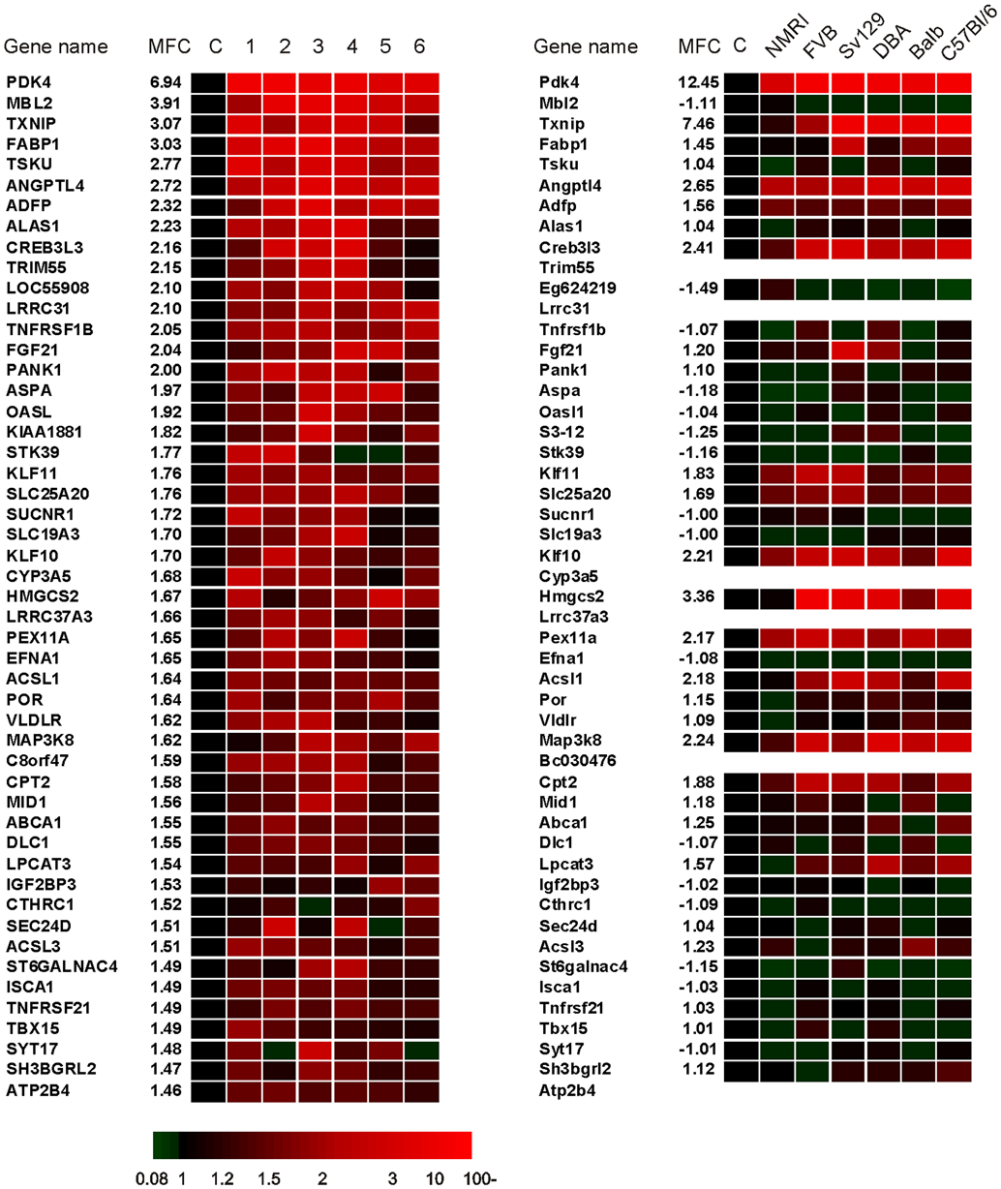
Partial conservation of Wy14643-induced gene regulation between human and mouse hepatocytes. Heat map illustrating the relative induction of the top 50 of upregulated genes in response to 6 h Wy14643 treatment in human hepatocytes. All genes were significantly changed (P<0.05) and were ranked based on mean fold-change (MFC). Expression levels in the DMSO-treated cells were set at 1. Relative changes in expression of the corresponding mouse orthologs in mouse hepatocytes are shown in parallel.

In order to identify additional genes that are specifically regulated by PPARα in one particular species, we performed scatter plot analysis comparing the effect of 6 h PPARα activation between mouse and human (Figure 5A). A similar plot was created for 24 h PPARα activation (Figure 5B). A number of genes could be identified that were induced by Wy14643 specifically in human (MBL2, CYP1A1, HMOX1 and TSKU) or mouse (Fbp2, Lgals4, Pxmp4 and Ucp2) . To directly compare the effect of Wy14643 on specific genes between mouse and human, genes that were upregulated by 6 h or 24 h Wy14643 in human hepatocytes were ranked according to their mean fold-change and the changes in expression compared between the individual donors (Figure 6 and Figure S2, respectively). The changes in expression of their mouse orthologs are presented in parallel. The picture clearly illustrates the human-specific induction of MBL2, ALAS1, TSKU, and many other genes. The specific induction of TSKU was confirmed by qPCR (Figure S3A). Interestingly, the top 50 of most highly induced genes contain a remarkably high number of established PPARα targets, regulation of which was conserved in mouse hepatocytes. This includes genes involved in mitochondrial fatty acid oxidation and ketogenesis (HMGCS2, CPT1A, CPT2, SLC25A20), peroxisomal/microsomal fatty acid oxidation (ECH1, CYP4A11), fatty acid binding and activation (FABP1, ACSL1, ACSL3), and lipid droplet associated proteins (ADFP). Wy14643 also stimulated expression of a number of secreted PPARα targets including FGF21 and ANGPTL4. These data support an important role for PPARα in the regulation of lipid metabolism in human hepatocytes. Besides numerous known PPARα target genes, several putative novel PPARα targets were found to be commonly regulated by Wy14643 in mouse and human, including the transcription factors CREB3L3, KLF10 and KLF11, and MAP3K8. Induction of KLF10 was confirmed by qPCR (Figure S3B).

**Figure 7:**
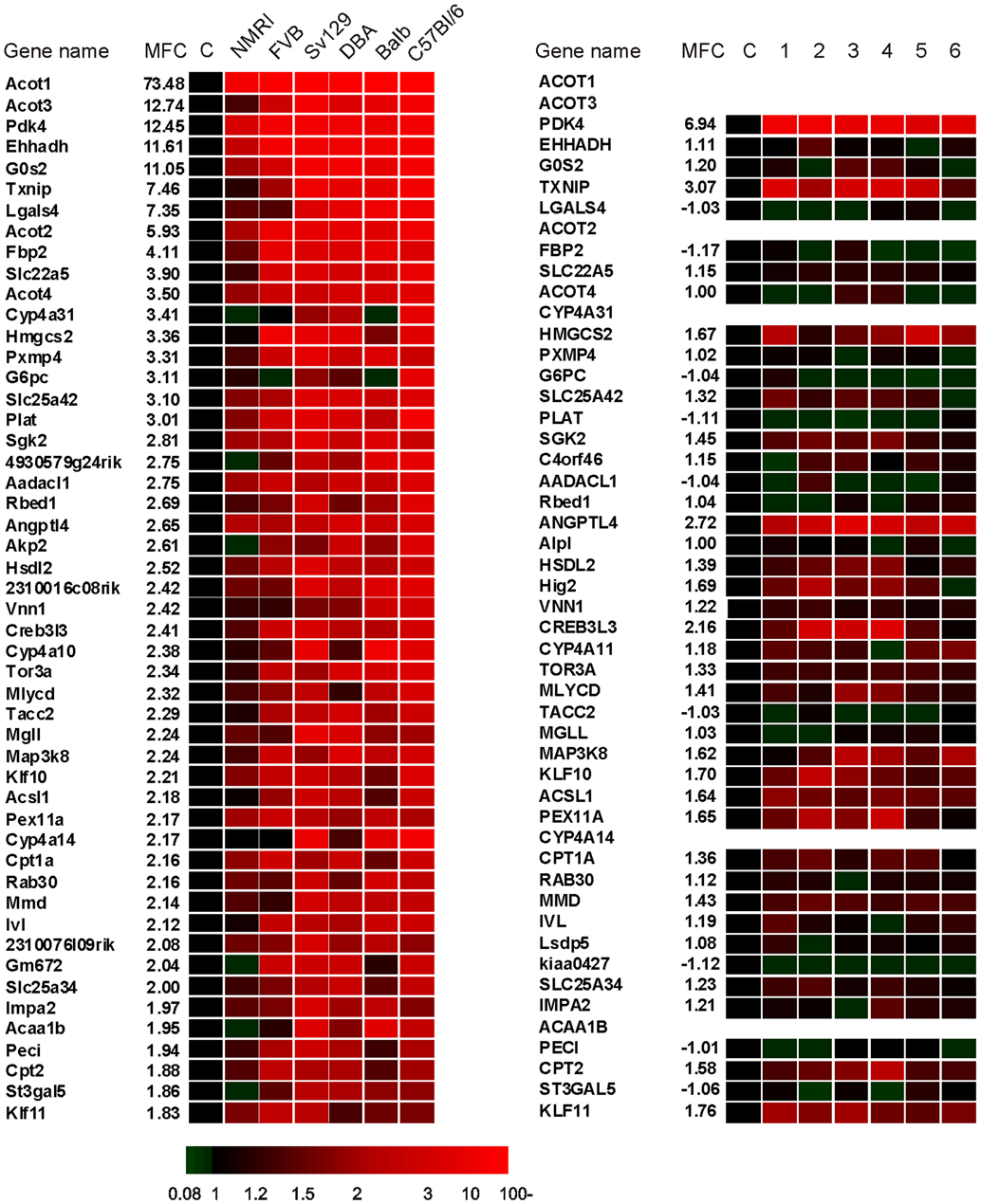
Partial conservation of Wy14643-induced gene regulation between mouse and human hepatocytes. Heat map illustrating the relative induction of the top 50 of upregulated genes in response to 6 h Wy14643 treatment in mouse hepatocytes. All genes were significantly changed (P<0.05) and were ranked based on mean fold-change (MFC). Expression levels in the DMSO-treated cells were set at 1. Relative changes in expression of the corresponding human orthologs in human hepatocytes are shown in parallel.

Conversely, the scatter plots and ranking of genes also clearly revealed numerous genes that were specifically induced by Wy14643 in mouse, including Fbp2, Lgals4, and Pxmp4, as well as known PPARα target genes such as Cd36, Cpt1b and Ucp2 (Figures 5B and 7 ; Figure S4). The mouse specific induction of Fbp2 was confirmed by qPCR (Figure S3C). These data suggest that in general the effect of PPARα activation is remarkably dissimilar between mouse and human hepatocytes. Nevertheless, many established PPARα targets representing key genes in lipid metabolism are commonly regulated in mouse and human.

### The role of PPARa in hepatic lipid metabolism is well conserved between mouse and human

**Figure 8:**
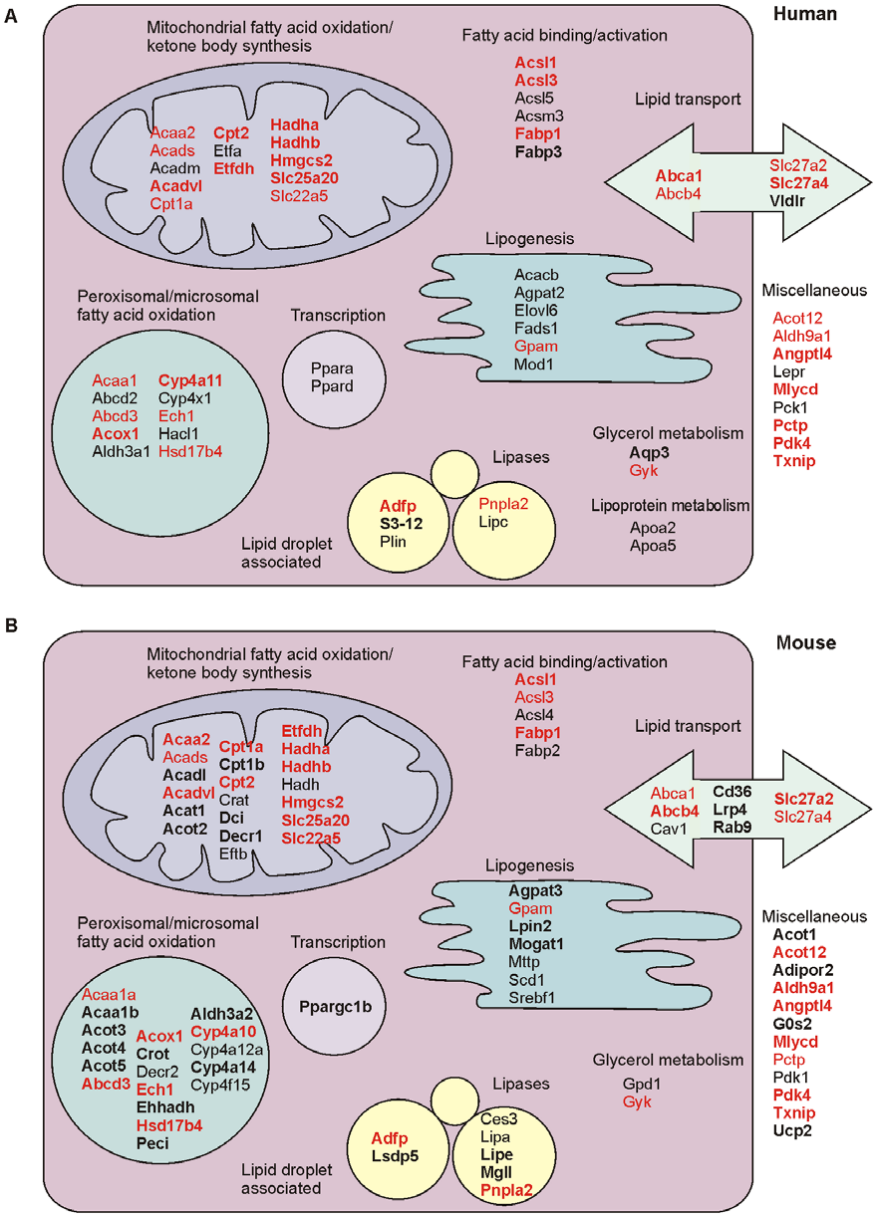
PPARα serves as a global transcriptional regulator of lipid metabolism in mouse and human hepatocytes. Genes significantly upregulated by Wy14643 and that function in lipid metabolism were classified into specific metabolic pathways. Separate pictures were created for human hepatocytes (A) and mouse hepatocytes (B). Genes significantly upregulated by Wy14643 at both time points of 6 h and 24 h are shown in bold. Genes significantly upregulated by Wy14643 in human and mouse hepatocytes are shown in red. Genes significantly upregulated by Wy14643 at one time point only are shown in normal font. Functional classification is based on a self-made functional annotation system of genes involved in lipid metabolism.

We showed that a large proportion of the genes commonly regulated by PPARα in mouse and human were involved in some aspect of lipid metabolism. To better appreciate the conservation of PPARα's role as master regulator of hepatic lipid metabolism, we classified genes according to specific lipid metabolic pathways to create a comprehensive picture of PPARα-dependent gene regulation (Figures 8A and B). The picture reveals that in human hepatocytes PPARα activation induces the expression of many genes involved in different aspects of lipid metabolism, including fat oxidation, fat synthesis, intracellular TG storage and hydrolysis, membrane transport, intracellular activation and trafficking of fatty acids and lipoprotein metabolism. Comparison with the corresponding picture for mouse reveals a remarkable conservation at the pathway level, indicating that the role of PPARα in hepatic lipid metabolism is highly similar between mice and human. The sole exception is lipoprotein metabolism, represented by APOA2 and APOA5, which was exclusively regulated in human hepatocytes. It is also evident that fewer peroxisomal genes are induced by Wy14643 in human vs. mouse hepatocytes. Interestingly, within a particular metabolic pathway the specific genes upregulated by Wy14643 to some extent differ between mouse and human. Taken together, the results suggest that in human and mouse hepatocytes PPARα has an equally important role in governing lipid metabolism with the exception of lipoprotein metabolism and to a lesser extent peroxisomal metabolism. However, the specific genes under control of PPARα in mouse and human are partially different.

## Discussion

Numerous studies have examined the effect of PPARα activation or deletion on hepatic gene regulation using transcriptomics. In general, these studies indicate that unlike many other nuclear receptors, PPARα governs the expression of a large set of genes, many of which are involved in fatty acid metabolism [22]–[27]. However, there has been no systematic comparison of the whole genome effects of PPARα activation in human versus mouse hepatocytes [28]. Accordingly, in the present paper we systematically compared the effect of PPARα activation in primary mouse and human hepatocytes using a whole genome transcriptomics approach. A number of important general conclusions can be drawn from our work. First, perhaps contrary to common conception, our data support a major role for PPARα in human liver, as evidenced by the large number of genes altered upon PPARα activation in primary human hepatocytes. Second, even though the human and mouse hepatocytes were not cultured under identical conditions, we feel comfortable to conclude that PPARα regulates a mostly divergent set of genes in mouse and human liver. For example, we found that metabolism of xenobiotics is specifically regulated by PPARα in human liver. Third, the role of PPARα as a master regulator of hepatic lipid metabolism is well conserved between mouse and human. However, within each lipid metabolic pathway the specific genes under control of PPARα in mouse and human differ to some extent.

In recent years, the role of PPARα in human liver has been questioned based on RNase protection data showing 10-fold lower levels of PPARα mRNA in human liver compared with mouse liver [19]. Additionally, human hepatoma HepG2 cells were shown to respond poorly to PPARα activation [21]. In contrast, we show by quantitative realtime PCR that in liver tissue and primary hepatocytes PPARα expression levels are similar between mouse and human. It is inherently difficult to compare hepatic PPARα expression between species as PPARα mRNA fluctuations throughout the day [29], is increased by fasting [30], and is reduced under conditions of inflammation [31]. Changes in PPARα expression will likely influence the transcriptional response to PPARα activation. Our comparative analysis of hepatic gene regulation by human PPARα vs. mouse PPARα should thus be considered an approximation. Despite the limitations, our analysis represents a major advancement in our understanding of PPARα function in human liver.

Consistent with a major role of PPARα in human hepatocytes, the number of genes significantly regulated by Wy14643 was very high and was similar to the number in mouse hepatocytes. Although induction of gene expression by Wy14643 was generally less robust in human hepatocytes, these cells likely lost some sensitivity due to the extended time between isolation and harvesting. We were able to exclude differences in cultured medium as an explanation for the lower fold-inductions in human hepatocytes (data not shown).

While the number of genes commonly regulated by PPARα in mouse and human hepatocytes may seem relatively small, which would suggest minor overlap in PPARα function between the two species, the overlap is more impressive when studied at the level of gene ontology. Many of the overlapping gene ontology classes represent pathways of lipid metabolism. Supporting these data, many of the 125 genes co-regulated by PPARα in mouse and human are involved in various aspects of hepatic lipid handling, including peroxisomal and mitochondrial fatty acid oxidation (ACOX1, ECH1), ketogenesis (HMGCS2), fatty acid binding and activation (FABP1, ACSL3), and fatty acid uptake (SLC27A2). Our analysis demonstrates that in human liver, analogous to the situation in mouse liver [26], PPARα serves as a global transcriptional regulator of lipid metabolism.

In addition to numerous established PPARα target genes, several genes were found to be co-regulated by Wy14643 in mouse and human that have not yet been linked to PPARα, including the liver specific transcription factor CREB3L3. CREB3L3 was recently shown to be involved in the hepatic acute phase response, suggesting that it may partially mediate the effects of PPARα on acute phase response [32]. Other conserved novel putative targets include MAP3K8, SGK2, and the transcription factor KLF10 and KLF11. KLF10 and KLF11 encode three zinc-finger Krüppel-like transcription factors that binds GCrich/Sp1-like sequences and influence cell proliferation [33].

The inability of PPARα agonists to induce peroxisome proliferation in human is well acknowledged, although the precise mechanism remains to be fully elucidated. Using humanized PPARα mice, it has been shown that the human PPARα receptor has the ability to induce peroxisome proliferation and peroxisomal fatty acid oxidation in the context of a mouse liver [34], [35]. However, in a previous study using HepG2 cells engineered to express PPARα at levels similar to mouse liver, ACOX1 and other peroxisomal genes were not induced by PPARα [36]. Similar results were obtained in primary human hepatocytes treated with fenofibrate [37]. In contrast, we find that a number of genes involved in peroxisomal fatty acid oxidation, including the prototypical PPARα targets ACOX1, ECH1, PEX11A, and ACAA1, is commonly induced by PPARα in mouse and human. Simultaneously, we find that induction by PPARα of numerous other peroxisomal genes, including Ehhadh, Pxmp4, Acot4, and Peci, is specific for mouse. Our data argue against a general mechanism and suggest that any lack of conservation of PPARα-dependent gene regulation between mouse and human must be determined at the level of individual target genes.

Previously, it was shown that APOA1, APOA2 and APOA5 are upregulated by PPARα agonists, which was found to be specific for humans [38]–[41]. While we confirm the human-specific upregulation of APO2 and APOA5 by Wy14643, we could not confirm the upregulation of APOA1 by Wy14643. Rather, we found a minor but statistically significant decrease in APOA1 expression after 6 h of Wy14643 treatment. The reason for this discrepancy is unclear but may be related to the type of PPARα agonist used. Overall, our data indicate that regulation of apolipoproteins A by PPARα is specific for humans, which very likely accounts for the human specific induction of plasma HDL levels by fibrates [42].

Several individual genes were identified that were also specifically regulated by Wy14643 in human hepatocytes. This includes the secreted mannose-binding lectin MBL2, which is an important protein of the humoral innate immune system [43], and TSKU, which encodes a secreted protein involved in development [44]. Regulation of CYP1A1 by PPARα in human hepatocytes has been previously observed [45], and was shown here to be part of a more comprehensive regulation of biotransformation enzymes by PPARα that was specific for human hepatocytes. Importantly, while genes belonging to the Cyp4a class are exclusively regulated by PPARα in mouse, genes belonging to CYP classes 1–3 are specifically regulated by PPARα in human, which confirms previous analyses [46], [47].

A number of pathways was found to be specifically induced by Wy14643 in mouse hepatocytes, including glycolysis/gluconeogenesis, pentose phosphate pathway, and glycerolipid metabolism, as were several specific lipid metabolic pathways. A similar mouse-specific response was observed at the level of individual genes. Most notable examples were Fbp2 (fructose-1,6-bisphosphatase 2), Lgals4 (lectin, galactoside-binding, soluble, 4), and several Acots (Acyl-CoA thioesterases).

Studies in mice have yielded considerable evidence for a direct role of PPARα in hepatic glucose metabolism. Importantly, fasted PPARα -/- mice exhibit markedly reduced plasma glucose levels [30]. Other studies have suggested a direct link between PPARα and hepatic gluconeogenesis [48]–[50]. In contrast, human trials generally do not support an effect of PPARα activation on plasma glucose levels. Accordingly, it is tempting to relate these seemingly discrepant results to the observed mouse-specific regulation of glucose metabolic pathways.

In conclusion, PPARα activation has a major impact on gene regulation in human liver cells. Importantly, the role of PPARα as a master regulator of hepatic lipid metabolism is generally well conserved between mouse and human. Overall, however, PPARα regulates a mostly divergent set of genes in mouse and human hepatocytes.

## Materials and Methods

### Materials

Wy14643 was obtained from ChemSyn Laboratories (Lenexa, KS). Recombinant human insulin (Actrapid) was from Novo Nordisk (Copenhagen, Denmark). SYBR Green was from Eurogentec (Seraing, Belgium). Fetal calf serum, penicillin/streptomycin/fungizone were from Lonza Bioscience (Verviers, Belgium). Otherwise, chemicals were from Sigma (Zwijndrecht, The Netherlands).

### Human primary hepatocytes

Human hepatocytes and Hepatocyte Culture Medium Bulletkit were purchased from Lonza Bioscience (Verviers, Belgium). Primary hepatocytes were isolated from surgical liver biopsies obtained from six individual donors who underwent surgery after informed consent was obtained for surgery with subsequent use of samples in experiments. Lonza utilizes the hospital's Institutional Review Board (IRB) to obtain approval before obtaining these tissues. The characteristics of the donors are presented in Table 1. Hepatocytes were isolated with two-step collagenase perfusion method and the viability of the cells was over 80%.

**Table 1:** Characteristics of the human liver donors.

Donors	Age (years)	Sex	Pathology
Donor 1	63	Male	Colo-rectal Metastasis
Donor 2	44	Male	Metastasis
Donor 3	70	Female	Hepatic metachrone lesion
Donor 4	54	Female	Metastasis
Donor 5	73	Male	Carcinome hepatocellular
Donor 6	73	Male	Endocrine carcinoma

Cells were plated on collagen-coated six-well plates and filled with maintenance medium. Upon arrival of the cells, the medium was discarded and was replaced by Hepatocyte Culture Medium (HCM) with additives. The additives included Gentamicin sulphate/Amphotercin-B, Bovine serum albumin (Fatty acid free), Transferrin, Ascorbic acid, Insulin, Epidermal growth factor, Hydrocortisone hemisuccinate. The next day, cells were incubated in fresh medium in the presence or absence of Wy14643 (50 µM) dissolved in DMSO for 6 and 24 hours, followed by RNA isolation.

### Mouse primary hepatocytes

Mouse hepatocytes were isolated by two-step collagenase perfusion as described previously [51] from 6 different strains of mouse; NMRI, SV129, FVB, DBA, BALB/C and C57BL/6J. The characteristics of the mice used are presented in Table 2.

**Table 2:** Characteristics of the mouse strains.

Mouse strain	Age (months)	Sex
NMRI	4	Male
BL/6	2	Male
Sv129	4	Male
FVB	5	Female
Balb/c	6	Male
DBA	3	Female

Cells were plated on collagen-coated six-well plates. Viability was determined by Trypan Blue exclusion, and was at least 75%. Hepatocytes were suspended in William's E medium (Lonza Bioscience, Verviers, Belgium) supplemented with 10% (v/v) fetal calf serum, 20 m-units/mL insulin, 10 nM dexamethasone, 100 U/mL penicillin, 100 µg/mL of streptomycin, 0.25 µg/mL fungizone and 50 µg/mL gentamycin. After four hours the medium was discarded and replaced with fresh medium. The next day, cells were incubated in fresh medium in the presence or absence of Wy14643 (10 µM) dissolved in DMSO for 6 and 24 hours, followed by RNA isolation. Isolation of mouse primary hepatocytes was approved by the animal ethics committee of Wageningen University. A 5-fold lower concentration of Wy14643 was used in mouse primary hepatocytes to take into account the higher affinity of Wy14643 for mouse PPARα compared to human PPARα [52].

### Affymetrix microarray

Total RNA was prepared from human and mouse primary hepatocytes using TRIzol reagent (Invitrogen, Breda, The Netherlands). RNA was used individually and further purified using RNeasy micro columns (Qiagen, Venlo, the Netherlands). RNA integrity was checked on an Agilent 2100 bioanalyzer (Agilent Technologies, Amsterdam, the Netherlands) using 6000 Nano Chips according to the manufacturer's instructions. RNA was judged as suitable for array hybridization only if samples exhibited intact bands corresponding to the 18S and 28S ribosomal RNA subunits, and displayed no chromosomal peaks or RNA degradation products (RNA Integrity Number>8.0). Five hundred nanograms of RNA were used for one cycle cRNA synthesis (Affymetrix, Santa Clara, CA). Hybridization, washing and scanning of Affymetrix Gene chip mouse genome 430 2.0 arrays (mouse primary hepatocytes) and human genome U133 2.0 plus was according to standard Affymetrix protocols.

Scans of the Affymetrix arrays were processed using packages from the Bioconductor project [53]. Expression levels of probe sets were calculated using GCRMA, followed by identification of differentially expressed probe sets using Limma. Comparison was made between treated and untreated (control) human primary hepatocyte, the same was compared for mouse primary hepatocyte. Probe sets that satisfied the criterion of *Raw P*<0.05 and a mean fold-change>±1.1 were considered to be significantly regulated. These selection criteria were based on careful inspection of the fold-changes in expression and their statistical significance of some known PPARα target genes, including Acadvl, Fatp4, and Acox1, which barely exceeded these thresholds. Functional analysis of the array data was performed by a method based on overrepresentation of Gene Ontology (GO) terms [54]–[56] and Gene Set Enrichment analysis [57]. Orthologs were retrieved via Homologene (NCBI). HomoloGene is a system for automated detection of homologs among the annotated genes of several completely sequenced eukaryotic genomes.

All Microarray data reported in the manuscript is described in accordance with MIAME guidelines.

### Q-PCR

1 µg of total RNA was reverse-transcribed with iScript (Bio-Rad, Veenendaal, the Netherlands). cDNA was PCR-amplified with Platinum Taq DNA polymerase (Invitrogen) on a Bio-Rad iCycler or MyIQ PCR machine. Primers were designed to generate a PCR amplification product of 100–200 bp and were taken from Primerbank (http://pga.mgh.harvard.edu/primerbank). Specificity of the amplification was verified by melt curve analysis and evaluation of efficiency of PCR amplification. The sequence of primers used are provided in Table S4. The mRNA expression of genes reported was normalized to universal 18S gene expression. To compare PPARα expression in mouse and human hepatocytes, primers were used that yielded amplicons of equal length. A standard curve was included to confirm amplification efficiency of 100%±2 for PPARα and for the 18S control gene. PPARα expression was calculated as 1/(2̂(CtPPARα a-Ct18S)), allowing for direct comparison between the two species.

Human liver RNA was obtained via Ambion and represented a mixture of RNA from 3 individuals without liver disease. Mouse liver RNA was obtained from 5 male mice on mixed genetic background (C57Bl/6-Sv129, fed state).

## Supporting Information

Figure S1 Principal component analysis illustrating the major sources of variation in our microarray dataset. In the first dimension, data separate based on species. The second dimension illustrates the effect of Wy14643 treatment.
(9.28 MB TIF)

Figure S2 Heat map illustrating the relative induction of the top 50 of upregulated genes in response to 24 h Wy14643 treatment in human hepatocytes. All genes were significantly changed (P<0.05) and were ranked based on mean fold-change (MFC). Expression levels in the DMSO-treated cells were set at 1. Relative changes in expression of the corresponding mouse orthologs in mouse hepatocytes are shown in parallel.
(10.38 MB TIF)

Figure S3 Species-specific induction of novel putative PPARα genes by Wy14643. (A) Relative induction of Kruppel-like factor 10 (KLF10) by Wy14643 in human and mouse hepatocytes. (B) Relative induction of Tukushin (TSKU) by Wy14643 in human and mouse hepatocytes. (C) Relative induction of fructose bisphosphatase 2 (Fbp2) by Wy14643 in mouse hepatocytes. Inductions for 6 h (grey bars) and 24 h (black bars) Wy14643 treatments are shown. Expression of cells treated with DMSO was set at 1 (white bars). Gene expression was determined by qPCR. Error bars represent SD. *P<0.05 according to Student's T-test.
(4.08 MB TIF)

Figure S4 Heat map illustrating the relative induction of the top 50 of upregulated genes in response to 24 h Wy14643 treatment in mouse hepatocytes. All genes were significantly changed (P<0.05) and were ranked based on mean fold-change (MFC). Expression levels in the DMSO-treated cells were set at 1. Relative changes in expression of the corresponding human orthologs in human hepatocytes are shown in parallel.
(10.30 MB TIF)

Table S1 Overlapping and species-specific list of significantly upregulated (1_1) and downregulated (1_2) genes in mouse and human primary hepatocytes after treatment with Wy14643 (6 h and/or 24 h). Genes were designated as significantly changed if satisfied the criteria of P<0.05 and mean fold change (MFC)>±1.1.
(0.24 MB XLS)

Table S2 Overlapping and species-specific list of Overrepresented Gene Ontology Classes in mouse and human primary hepatocytes after treatment with Wy14643 (6 h and/or 24 h), based on a functional class score method, FDR<0.0001. GO classes in bold font are species specifically overrepresented.
(0.06 MB XLS)

Table S3 Overlapping and species-specific list of induced gene sets in mouse and human primary hepatocytes after treatment with Wy14643 (6 h and/or 24 h), gene sets included between 15 and 250 genes and FDR<0.25.
(0.02 MB XLS)

Table S4 Sequences of primer pairs used.
(0.03 MB DOC)
