# Supplementary figures and images for: Comparative Analysis of Gene Regulation by the Transcription Factor PPARα between Mouse and Human
Source: PLoS One. 2009 Aug 27;4(8):e6796. doi: 10.1371/journal.pone.0006796 (PMC2729378; doi:10.1371/journal.pone.0006796)

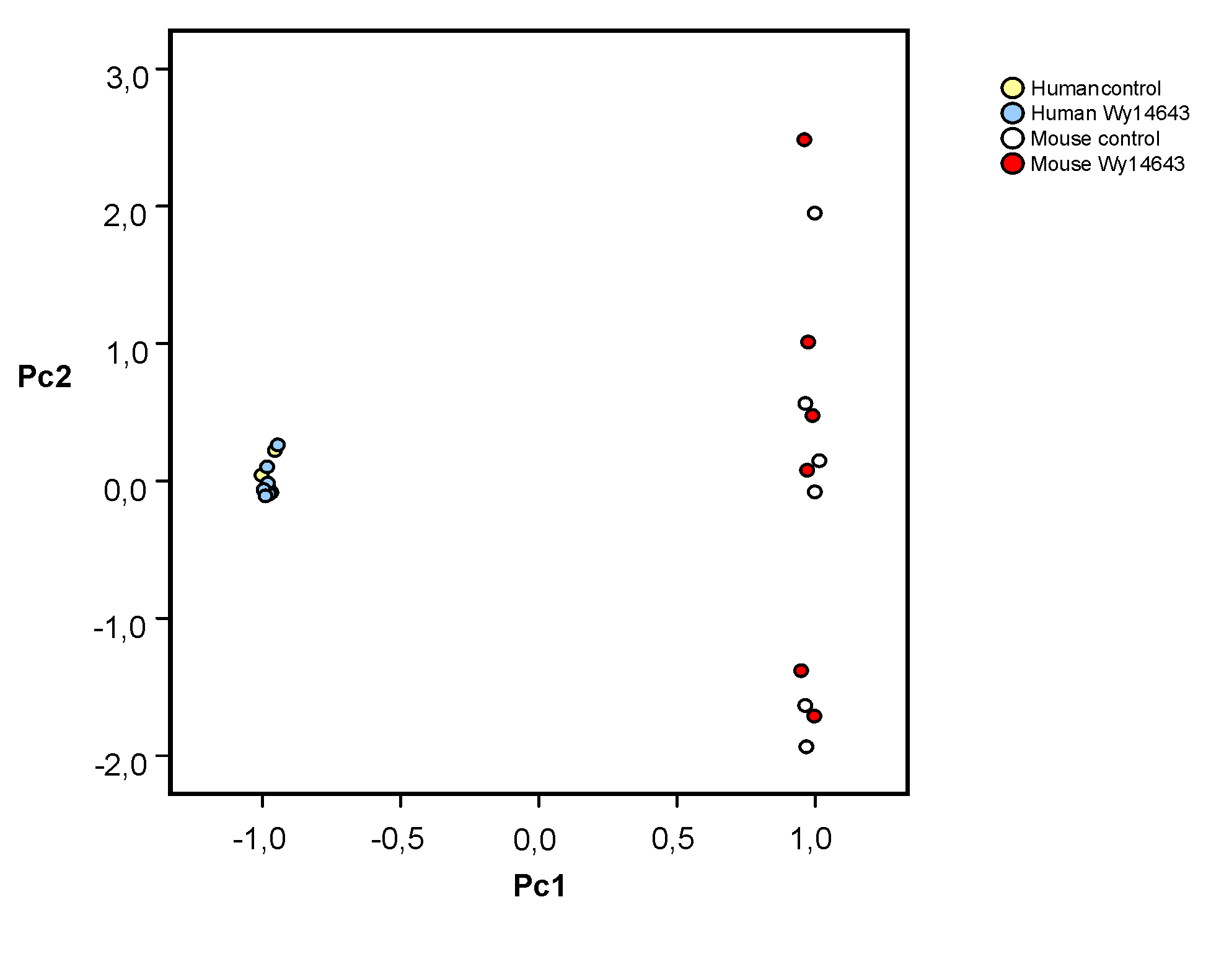

Supplement: Figure S1 — Principal component analysis illustrating the major sources of variation in our microarray dataset. In the first dimension, data separate based on species. The second dimension illustrates the effect of Wy14643 treatment. (9.28 MB TIF) [file pone.0006796.s001.tif]

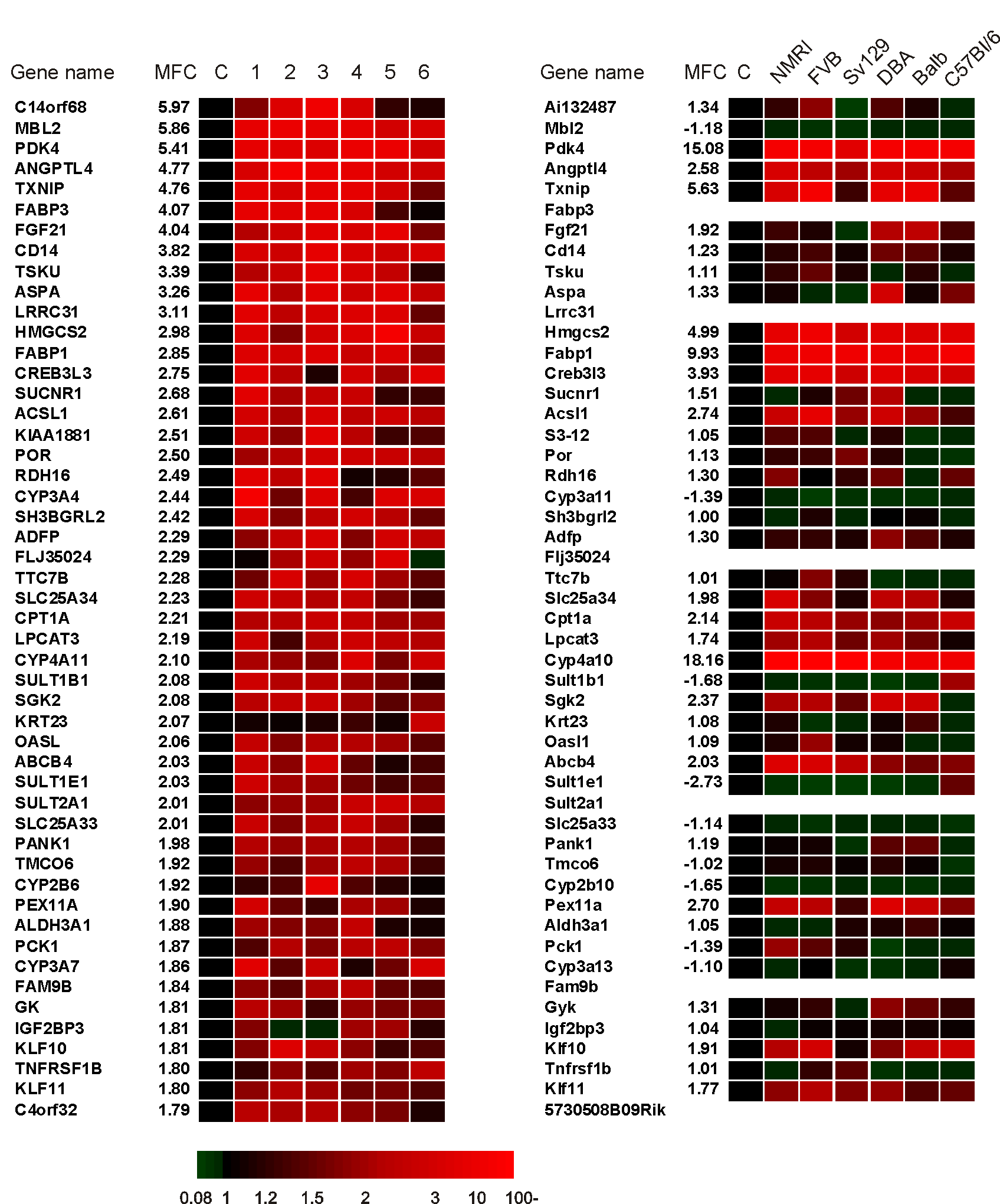

Supplement: Figure S2 — Heat map illustrating the relative induction of the top 50 of upregulated genes in response to 24 h Wy14643 treatment in human hepatocytes. All genes were significantly changed (P<0.05) and were ranked based on mean fold-change (MFC). Expression levels in the DMSO-treated cells were set at 1. Relative changes in expression of the corresponding mouse orthologs in mouse hepatocytes are shown in parallel. (10.38 MB TIF) [file pone.0006796.s002.tif]

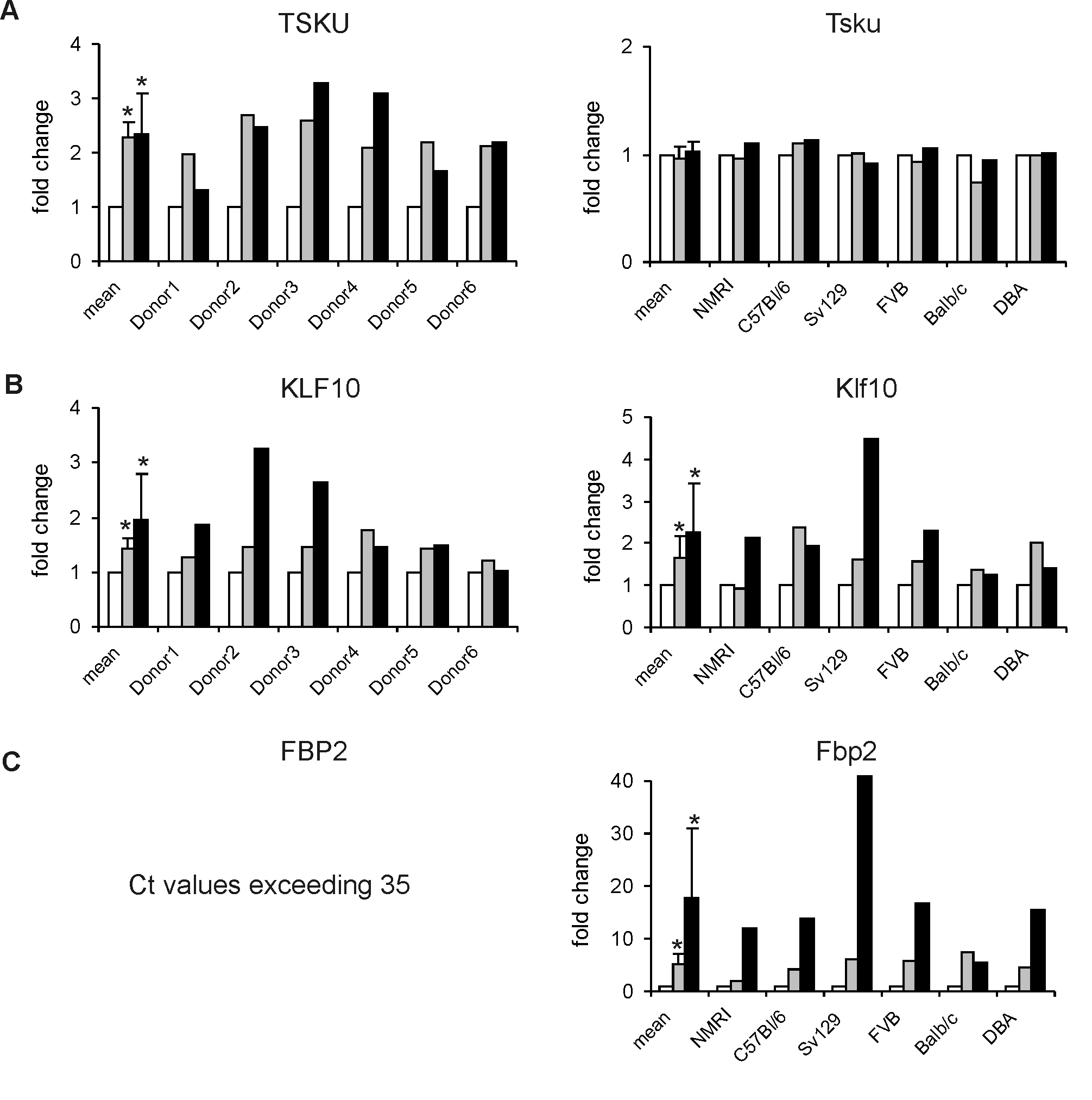

Supplement: Figure S3 — Species-specific induction of novel putative PPARα genes by Wy14643. (A) Relative induction of Kruppel-like factor 10 (KLF10) by Wy14643 in human and mouse hepatocytes. (B) Relative induction of Tukushin (TSKU) by Wy14643 in human and mouse hepatocytes. (C) Relative induction of fructose bisphosphatase 2 (Fbp2) by Wy14643 in mouse hepatocytes. Inductions for 6 h (grey bars) and 24 h (black bars) Wy14643 treatments are shown. Expression of cells treated with DMSO was set at 1 (white bars). Gene expression was determined by qPCR. Error bars represent SD. *P<0.05 according to Student's T-test. (4.08 MB TIF) [file pone.0006796.s003.tif]

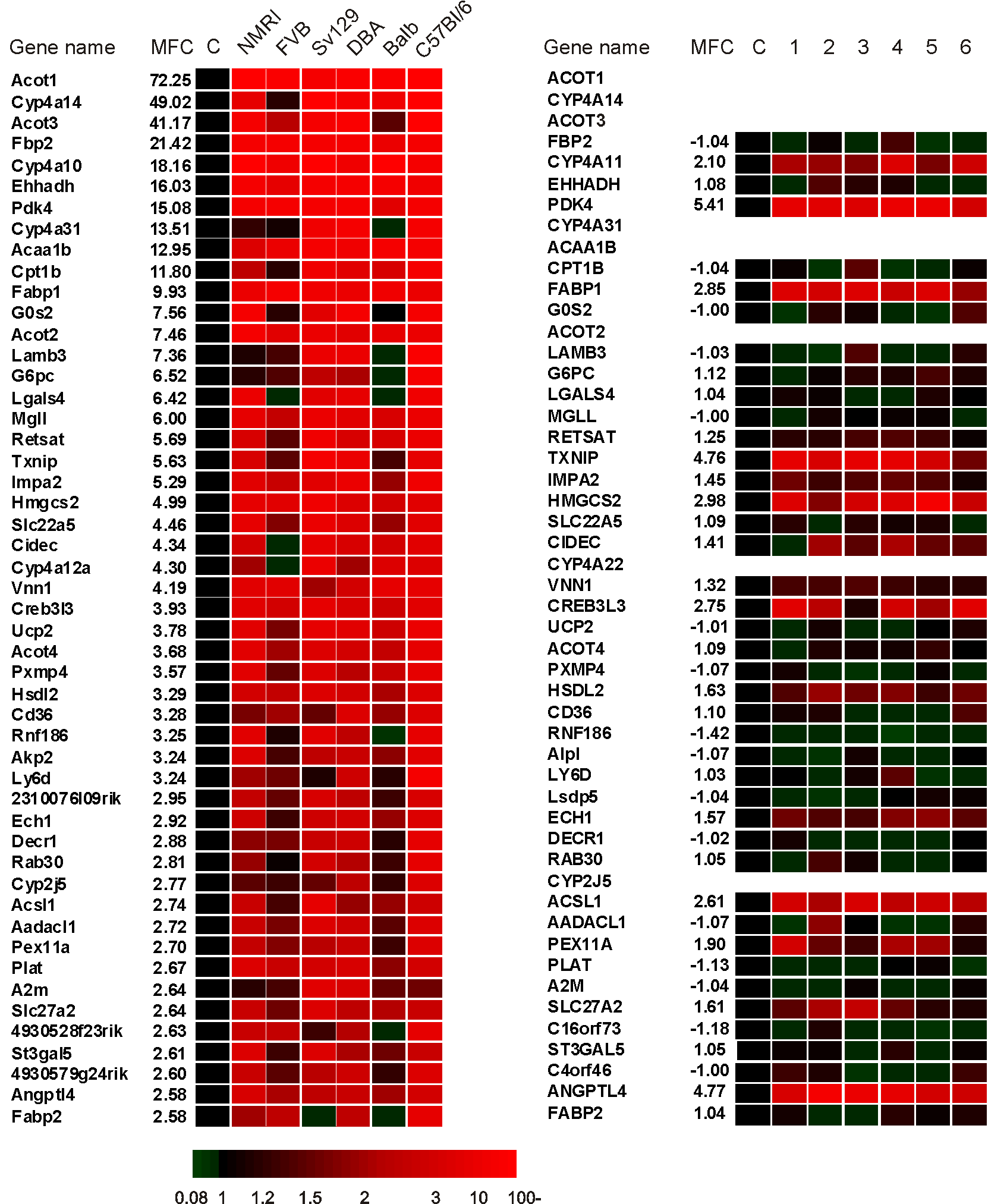

Supplement: Figure S4 — Heat map illustrating the relative induction of the top 50 of upregulated genes in response to 24 h Wy14643 treatment in mouse hepatocytes. All genes were significantly changed (P<0.05) and were ranked based on mean fold-change (MFC). Expression levels in the DMSO-treated cells were set at 1. Relative changes in expression of the corresponding human orthologs in human hepatocytes are shown in parallel. (10.30 MB TIF) [file pone.0006796.s004.tif]
