# Supplementary material for: Comparative Analysis of Gene Regulation by the Transcription Factor PPARα between Mouse and Human
Source: PLoS One. 2009 Aug 27;4(8):e6796. doi: 10.1371/journal.pone.0006796 (PMC2729378; doi:10.1371/journal.pone.0006796)
Supplement: Table S4 — Sequences of primer pairs used. (0.03 MB DOC) [file pone.0006796.s008.doc]

Supplementary Table 4 Primer sequences used for qPCR

Gene *Forward primer* *Reverse primer*

hPPARα CAGAACAAGGAGGCGGAGGTC TTCAGGTCCAAGTTTGCGAAGC

mPparα TAT TCG GCT GAA GCT GGT GTAC CTG GCA TTT GTT CCG GTT CT

hCPT1 TCCAGTTGGCTTATCGTGGTG CTAACGAGGGGTCGATCTTGG

mCpt1a CTC AGT GGG AGC GAC TCT TCA GGC CTC TGT GGT ACA CGA CAA

hPDK4 TGGAGCATTTCTCGCGCTAC ACAGGCAATTCTTGTCGCAAA

mPdk4 CCGCTTAGTGAACACTCCTTC TCTACAAACTCTGACAGGGCTTT

hTSKU CCTGGAGTCGCTTGACCTC CTGGTTGTGGCTAAGGTTCAC

mTsku TTC CAACCGGCTAGAAACCG GAGAAGGCAGAGGGCATGAT

hKLF10 TGCCAAACCTCACATTGCC GTATGACGAATCACACTTGTTGC

mKlf10 GGTGTCAAGTGCTTCTGCAA GAACTGAGCCCTGTCCTCTG

hFBP2 ACCCTGACCCGCTACGTTAT ACGCTTCCTGCGATTCCATAC

mFbp2 GGGGGAAATATGTGGTTTGCT TCCTCCGTGGTCTTTCTGTAAA

uni18S CGGCTACCACATCCAAGGA CCAATTACAGGGCCTCGAA
